# Electroformation of Giant Unilamellar Vesicles from Damp Lipid Films Formed by Vesicle Fusion

**DOI:** 10.3390/membranes13030352

**Published:** 2023-03-18

**Authors:** Zvonimir Boban, Ivan Mardešić, Sanja Perinović Jozić, Josipa Šumanovac, Witold Karol Subczynski, Marija Raguz

**Affiliations:** 1 Department of Medical Physics and Biophysics, University of Split School of Medicine, 21000 Split, Croatia; zvonimir.boban@mefst.hr (Z.B.); imardesi@mefst.hr (I.M.); 2 Faculty of Science, University of Split, Doctoral Study of Biophysics, 21000 Split, Croatia; 3 Department of Organic Technology, Faculty of Chemistry and Technology, University of Split, 21000 Split, Croatia; 4 Department of Physics, Faculty of Science, University of Split, 21000 Split, Croatia; 5 Department of Biophysics, Medical College of Wisconsin, Milwaukee, WI 53226, USA; subczyn@mcw.edu

**Keywords:** GUV, electroformation, cholesterol, damp lipid film, rapid solvent exchange, plasma cleaning, cholesterol crystals, vesicle fusion

## Abstract

Giant unilamellar vesicles (GUVs) are artificial membrane models which are of special interest to researchers because of their similarity in size to eukaryotic cells. The most commonly used method for GUVs production is electroformation. However, the traditional electroformation protocol involves a step in which the organic solvent is completely evaporated, leaving behind a dry lipid film. This leads to artifactual demixing of cholesterol (Chol) in the form of anhydrous crystals. These crystals do not participate in the formation of the lipid bilayer, resulting in a decrease of Chol concentration in the bilayer compared to the initial lipid solution. We propose a novel electroformation protocol which addresses this issue by combining the rapid solvent exchange, plasma cleaning and spin-coating techniques to produce GUVs from damp lipid films in a fast and reproducible manner. We have tested the protocol efficiency using 1/1 phosphatidylcholine/Chol and 1/1/1 phosphatidylcholine/sphingomyelin/Chol lipid mixtures and managed to produce a GUV population of an average diameter around 40 µm, with many GUVs being larger than 100 µm. Additionally, compared to protocols that include the dry film step, the sizes and quality of vesicles determined from fluorescence microscopy images were similar or better, confirming the benefits of our protocol in that regard as well.

## 1. Introduction

Liposomes are commonly used by researchers investigating membrane properties in a controlled environment. Based on their structure, we classify vesicles into unilamellar, multilamellar, and oligolamellar vesicles. Unilamellar vesicles only have a single outer bilayer, multilamellar vesicles contain multiple bilayers arranged in concentric circles, and oligolamellar vesicles enclose smaller ones inside. Unilamellar vesicles are further sorted by size into small (<100 nm), large (100 nm–1 µm), and giant unilamellar vesicles (GUVs, >1 µm). Small and large unilamellar vesicles are more often studied in the context of drug delivery applications and GUVs are more interesting to researchers studying membrane properties and organization because of the similarity in size to eukaryotic cells [1]. An additional advantage of GUV size is the possibility to observe them using light microscopy techniques.

Historically, GUVs were first produced using the natural swelling method introduced by Reeves and Dowben in 1969 [2]. Using this method, the vesicles are formed primarily due to osmotic pressure driving the aqueous solution in between the stacked lipid bilayers, causing them to close up into vesicles. However, the proportion of GUVs that can be generated using this method is small, as most of them are either multilamellar or display other types of defects [3].

Nowadays, one of the most commonly used methods for production of GUVs is electroformation, which facilitates the production of vesicles by applying an electric field to the lipid film [4]. Briefly, the lipids dissolved in an organic solvent are deposited onto the electrode. The organic solvent evaporates, and the remaining traces are vacuumed away, leaving a dry lipid film on the electrode surface. The coated electrode is used to construct a chamber which is then filled with an internal solution of choice and connected to an alternating current generator. Film hydration aided by the influence of an external electric field detaches the lipids from the surface, producing vesicles which can be observed under a microscope [5]. Compared to vesicles grown using the gentle hydration method, the electroformation method reduces the compositional heterogeneity of the vesicles [6] and increases the proportion of unilamellar vesicles [3]. The method has evolved significantly over the years, with many potential pitfalls identified and protocol modifications tested [7,8,9]. The most important issues are related to the use of organic solvents during lipid film deposition, reproducibility of the conventional film deposition technique, and the step in which the lipid film is completely dried.

Researchers tried to replace the organic solvent with an aqueous solution during the film deposition step [10,11]. They concluded that using aqueous solutions improved the efficiency of GUV formation in water as well as in buffers at physiologically relevant concentrations [10,12]. This was attributed to the ability of aqueous dispersions to produce well-oriented membrane stacks on the electrode [10]. Additionally, removal of organic solvents from the process should be beneficial for protocols dealing with protein reconstitution into GUVs due to reduced protein denaturation [12,13,14,15,16].

Regarding the lipid film deposition step, most electroformation protocols still use the drop-deposition method for preparation of the lipid film [4,8]. However, that approach results in films of nonuniform thickness [17]. Consequently, GUVs with a wide size distribution and different compositions are created and the experiment reproducibility is very low. Over the years, multiple attempts have been made to address this issue [8,17,18,19,20]. One of these was a study by Estes and Mayer who tested lipid film deposition using the spin-coating method [17]. The lipid solution is dropped onto a flat indium-tin oxide (ITO) coated glass surface, which is subsequently spun at a large angular velocity in order to obtain a film of uniform thickness. The uniformity of the lipid film and method reproducibility were validated by ellipsometry and atomic force microscopy. The method has been accepted by several groups using a wide range of lipid compositions to produce GUVs [17,21,22,23,24].

Another issue is the dry film step of the traditional protocol. This step creates a problem when working with lipid solutions containing high amounts of cholesterol (Chol). In such situations, some Chol demixes and forms anhydrous Chol crystals [11,25,26]. Once the film is rehydrated, these crystals do not participate in the formation of the lipid bilayer, resulting in an artifactual decrease of Chol concentration in the bilayer compared to the initial mixing ratio in the lipid solution. An example of Chol demixing was described in a study which utilized confocal microscopy to detect pure Chol bilayer domains in GUVs formed from a mixture of Chol and 1-palmitoyl-2-oleoyl-glycero-3-phosphocholine (POPC) using the traditional electroformation method (with the dry lipid film step) [26]. Chol bilayer domains were observed only for about 75 mol% of Chol in the initial mixture (Chol/POPC mixing ratio of 3/1), and not at 50 mol% as expected.

A method called the rapid solvent exchange (RSE) can be utilized to bypass the dry phase. During the procedure, chloroform-dissolved lipids are first mixed with an aqueous medium and the chloroform is then rapidly evaporated from the mixture [27]. The method has proven effective against the Chol demixing artifact [11,25,28]. However, it results in the formation of smaller multilamellar vesicles (MLVs), not GUVs. Paramagnetic resonance measurements on MLVs produced using the RSE method showed that pure Chol bilayer domains start to form at 50 mol% of Chol (at a Chol/phospholipid molar ratio of 1/1) [28,29]. Comparison of the amount of Chol in the initial lipid mixture needed for detection of Chol bilayer domains in RSE MLVs and GUVs produced with the conventional electroformation protocol attests to the severity of Chol demixing during the lipid film drying step.

Baykal-Caglar et al. have attempted electroformation from damp lipid films obtained by depositing an aqueous RSE-produced solution of MLVs onto the electrode and then slowly drying it under high-humidity conditions [11]. Their results show a decrease in the average transition temperature of GUVs made from damp compared to dry lipid films, implying a higher Chol concentration in GUVs made from the damp film. The main disadvantage of their approach is the long preparation time due to prolonged drying (22–25 h) in high-humidity conditions. Additionally, the obtained lipid film would inevitably display nonuniformities due to using the drop-deposition technique for the deposition of the suspension of MLVs.

Advancements to the traditional protocol have been proposed regarding the electrode-cleaning approaches as well. Traditionally, electrodes are cleaned prior to film deposition by applying organic solvents and then drying them. Plasma cleaning has also been tried out on ITO glass as an alternative and has proved to be very effective [30]. Moreover, treating the electrodes with plasma has enabled researchers to efficiently produce GUVs containing buffers with physiological levels of charged particles, which was very hard to achieve using conventional protocols [30]. The improvement has been attributed to easier hydration of the lipid film and subsequent formation of lipid bilayers [30]. However, this experiment used plasma treatments only as a method for electrode cleaning, and traditional film deposition which uses organic solvents was used in the protocol.

In this article, we introduce a novel electroformation protocol which includes the most useful modifications to the traditional protocol and combines them in a novel way in order to bypass the dry film phase. As far as we know, no one before tried to produce lipid films by depositing an aqueous suspension of liposomes on plasma-cleaned surfaces. Our approach was inspired by the vesicle fusion method which is often used for preparation of supported bilayer membranes [31]. The method involves the deposition of an aqueous suspension of vesicles on a hydrophilized surface. The interaction of the hydrophilic surface with the vesicles causes them to rupture, creating a surface bilayer.

Compared to the approach used by Baykal-Caglar et al., the new protocol significantly reduces the preparation time and increases the experiment reproducibility. Consequently, amongst other benefits, the protocol improves the electroformation of GUVs with higher Chol concentrations. Such GUVs are interesting to researchers investigating the role of Chol in fiber cell plasma membranes of the eye lens [26,32,33] or the development of atherosclerosis [34,35]. Moreover, bypassing the dry state results in a protocol more compatible with protein insertion into GUVs [13]. The advantage of such protocols is reduced protein denaturation, which occurs when preparing GUVs from lipids dissolved in an organic solvent or during film drying.

## 2. Materials and Methods

### 2.1. Materials

1-palmitoyl-2-oleoyl-glycero-3-phosphocholine (POPC), egg sphingomyelin (SM), and Chol were obtained from Avanti Polar Lipids Inc. (Alabaster, AL, USA). The fluorescent dye 1,1-dioctade-cyl-3,3,3,3-tetramethylindocarbocyanine Perchlorate (DiIC_18_(3)) was purchased from Invitrogen, Thermo Fisher Scientific (Waltham, MA, USA). When not used, the lipids were stored at −20 °C. The purity of chloroform (BDH Prolabo) was greater than 99.8%. ITO glass (ICG-90 INS 115, resistance 70–100 Ω) was purchased from Delta Technologies (Loveland, LO, USA). ITO glass dimensions were 25 × 75 × 1.1 mm. New ITO glass was used for each preparation in order to prevent coating deterioration [36]. Mili-Q (Merck, Rahway, NJ, USA) deionized water preheated to 60 °C was used as the internal chamber solution.

### 2.2. Preparation of the Suspension of Large Unilamellar Vesicles

MLVs were first prepared using a home-built RSE device to bypass the dry phase and the Chol demixing artifact. The chloroform dissolved lipid mixture was produced from 25 mg/mL POPC, 25 mg/mL SM, 20 mg/mL Chol, and 1 mg/mL DiIC_18_(3) stocks. The POPC/Chol molar ratio was 1/1 and the POPC/SM/Chol ratio 1/1/1. The molar ratio of the fluorescent probe with respect to POPC was 1/500. The total lipid mass was 2.1 mg. This resulted in an organic solution of lipids with a volume of 94.4 µL for the POPC/Chol mixture and 90.4 µL for the POPC/SM/Chol mixture. A volume of 400 µL Mili-Q deionized water was then added to the solution and the resulting mixture was vortexed (Vortex IR, Star Lab, Blakelands, UK) at a velocity of 2200 rpm. After initiating the vortexing, the pressure was slowly decreased to a approximately 0.05 bar using a vacuum pump (HiScroll 6, Pfeiffer Vacuum, Asslar, Germany). After reaching the desired pressure, the vortexed sample was kept under vacuum for an additional 90 s. The obtained suspension of MLVs was extruded using an Avanti Mini Extruder (Avanti Polar Lipids, Inc, Alabaster, AL.). The suspension was passed through a 100 nm polycarbonate (Nuclepore Track-Etch Membrane, Whatman, UK) filter 15 times in order to obtain a homogeneous large unilamellar vesicle (LUV) suspension. In order to prevent the loss of lipids during the initial wetting of the filtering segment, before extruding the suspension, deionized water was passed through the filter to pre-wet the extruder parts. Finally, extra water was added in order to achieve a final lipid concentration of 3.5 mg/mL.

### 2.3. Preparation of the Damp Lipid Film

Prior to spin-coating, the ITO glass was immersed in deionized water for at least 45 min before being wiped four times with 70% ethanol moistened lint-free wipes. The glass was then plasma-cleaned with oxygen for 1 min using a plasma cleaner (PDC-002-HPCE with the PLASMAFLO PDC-FMG-2 attachment, Harrick Plasma, Ithaca, NY, USA) attached to a vacuum pump (HiScroll 6, Pfeiffer Vacuum, Assler, Germany).

Following that, 550 µL of the LUV suspension was deposited onto the electrode and spin-coated using a spin-coater (SM-150, Sawatec, Sax, Switzerland) to obtain the damp lipid film. The glass was spun at 600 rpm with the final velocity reached in 1 s. In order to prevent any unwanted evaporation, following spin-coating, the coated ITO glass was placed in a Petri dish and immediately used for the assembly of an electroformation chamber.

### 2.4. Electroformation Protocol

The electroformation chamber is made of two 25 × 37.5 mm ITO-coated glass electrodes separated by a 1.6 mm thick teflon spacer. The electrodes were made by cutting a 25 × 75 mm ITO glass slide in half using a diamond pen cutter. After lipid deposition, the chamber was assembled by attaching the spacer to the electrodes using vacuum grease. Upon insertion, the stopper was also sealed with vacuum grease. This way, contact between the grease and the internal solution is avoided, minimizing the possibility of harmful effects due to grease contamination [28]. The structure of the chamber is further secured by binding clips attached at three points on the electrodes—two next to the stopper, and one at the opposite side. Finally, the chamber was attached to a pulse generator (UTG9005C, UNI-T, Dongguan City, China or PSG 9080, Joy-IT, Neukirchen-Vluyn, Germany) and placed inside an incubator at a temperature of 60 °C. In order to assure good contact between the conductor wires and the electrodes, the outer edges of the electrodes were covered with copper tape. Based on experience from our previous electroformation studies [21,22], the electrical parameters were set to 2 V and 10 Hz. After 2 h, the voltage was turned off and the chamber was kept in the incubator for another hour.

### 2.5. Fourier Transform Infrared Spectroscopy

Attenuated total reflection Fourier transform infrared spectroscopy (ATR-FTIR) was used to obtain spectra of solutions before and after the RSE procedure, and spectra of glass slides before and after spin-coating. We used the Spectrum Two (Perkin-Elmer, Waltham, MA, USA) spectrometer.. The scans were performed for wavenumbers ranging from 4000 to 450 cm^−1^ at a resolution of 4 cm^−1^ in 5 scans at 25 °C. A diamond was used as the reflection crystal. The obtained spectra were compared with each other and with the reference spectra in the Spectrum IR library to confirm the composition of the samples.

### 2.6. Fluorescence Imaging and Data Analysis

In order to search the entire volume of the chamber, we collected images from 13 regions on the sample. One hundred vesicles were randomly chosen from the images. If the images did not contain 100 vesicles, all observed vesicles were tracked. Images were obtained using a fluorescence microscope (Olympus BX51, Olympus, Tokyo, Japan). Vesicle diameters were measured using the line tool in the Fiji software [37].

### 2.7. Dynamic Light Scattering

The measurement of the hydrodynamic diameter and polydispersity index of liposome suspensions was performed using dynamic light scattering (Litesizer 500, Anton Paar, Graz, Austria). For the measurements, 100 µL of the liposome suspension was mixed with 900 µL of phosphate buffer.

### 2.8. Data Analysis

If not explicitly stated otherwise, numerical results are expressed as mean ± standard deviation. Sample distribution normality was assessed visually through histograms and formally by using the Shapiro–Wilk test. Depending on whether or not the normality assumption was violated, the difference of means for two groups was tested using the Student’s t-test or the Wilcoxon rank sum test. All data analysis and visualization was performed using the R programming language [38].

## 3. Results and Discussion

### 3.1. The New Protocol

**Figure 1:**
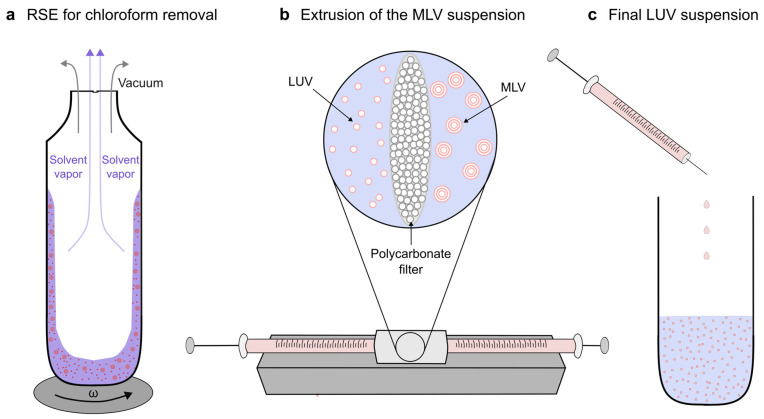
Preparation of the LUV solution. (**a**) MLVs are first prepared using the RSE method by rapidly evaporating chloroform from the mixture. (**b**) MLVs are passed through a polycarbonate filter an uneven number of times in order to obtain LUVs. (**c**) The obtained LUV solution is stored for later use in preparation of the damp lipid film.

We present a novel protocol for production of GUVs from damp lipid films. In order to bypass the dry phase, an aqueous suspension of MLVs is obtained using the RSE method (Figure 1a) and then passed through an extruder in order to obtain a population of large unilamellar vesicles (LUVs) (Figure 1b,c).

**Figure 2:**
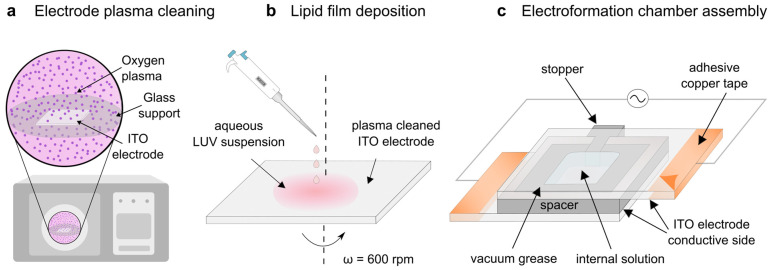
Electroformation from a damp lipid film. (**a**) The ITO electrode is hydrophilized using a plasma cleaner. (**b**) The LUV suspension is deposited onto a plasma cleaned ITO coated glass and spin-coated to obtain a damp lipid film. (**c**) The coated electrode is used to assemble the electroformation chamber.

The LUV suspension is deposited onto an indium tin oxide (ITO) coated glass which was previously plasma-cleaned, making the surface hydrophilic (Figure 2a,b). The interaction of the vesicle bilayers with the hydrophilic surface causes them to rupture during spin-coating, leaving behind a thin damp lipid film on the electrode. This electrode is then used in construction of the electroformation chamber which is subsequently placed in an incubator and connected to an alternating current source in order to form GUVs (Figure 2c).

**Figure 3:**
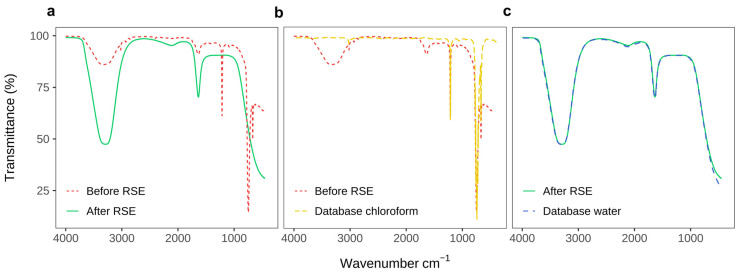
Confirmation of chloroform removal by the RSE method from a mixture of water and chloroform dissolved lipids. (**a**) Comparison of FTIR spectra before and after performing RSE. (**b**) FTIR spectrum before RSE compared to the database curve for chloroform. (**c**) FTIR spectrum of a sample after performing the RSE method compared with the database spectrum of water.

We have confirmed that chloroform has successfully been removed from the solution using ATR-FTIR spectroscopy (Figure 3a). Compared to the solution containing chloroform, after applying the RSE method, there are no absorption peaks at around 700 and 1200 cm^−1^ due to CCl_3_-stretching and CH-bending in the fingerprint region of the spectrum (Figure 3a,b), respectively. The remaining signal corresponds to the water spectrum from the database (Figure 3c), with characteristic peaks around 3400 and 1650 cm^−1^. 

**Figure 4:**
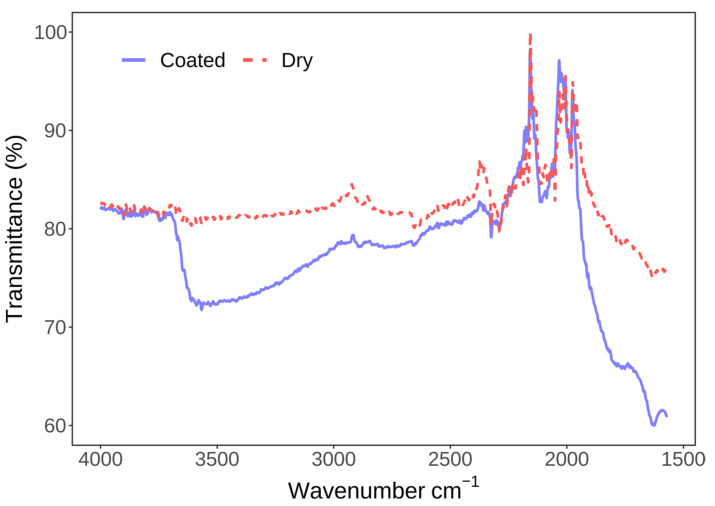
Confirmation of water presence before (red) and after (blue) one minute of water spin-coating on coverslip glass. Aside from the water peaks, there are also additional absorption peaks due to the diamond and glass absorption bands.

In order to prove that the film remained damp after spin-coating, we have also compared ATR-FTIR spectra of glass before and after spin-coating (Figure 4). The blue curve, representing the sample after spin-coating, displays an additional broad peak around 3400 cm^−1^ due to the O-H stretching vibrations and another one around 1650 cm^−1^ corresponding to absorption due to H-O-H bending, confirming the presence of water. Both curves display multiple peaks between 2300 and 1900 cm^−1^ representing the absorption bands of diamond. In this region, diamond does not have full transmission capability, and the bands from the ATR reflection crystal are visible. The downward slope in the region from 2000 to 1500 cm^−1^ appears due to the glass on which the coating was performed.

This confirms that our approach avoids the dry film phase just like that of Baykal-Caglar et al., so it should also result in increased compositional homogeneity of the GUVs and reduction of the demixing artefact [11]. The great advantage of our protocol is a significant decrease in preparation time (22–25 h of drying compared to spin-coating, which lasts up to a couple of minutes) and the potential to create more homogeneous lipid films. Furthermore, aside from inducing vesicle rupture, treating the electrodes with plasma has also been proven beneficial for electroformation efficiency, enabling the production of GUVs with charged lipids and solutions containing high ion concentrations. The effect has been attributed to easier hydration of the lipid film and subsequent formation of lipid bilayers [30].

### 3.2. The Effect of Spin-Coating Duration

To demonstrate the efficiency of the new protocol, we produced GUVs from a 1/1 POPC/Chol molar ratio lipid mixture. The hydrodynamic diameter of the RSE-produced MLVs was 1401.2 nm with a polydispersity index of 0.3. After extrusion, the diameter of obtained LUVs was 136 nm with a polydispersity index of 0.11. The observation that the average size of LUVs is larger than the 100 nm pore size of the polycarbonate filter can be attributed to elastic deformations of LUVs [39].

**Figure 5:**
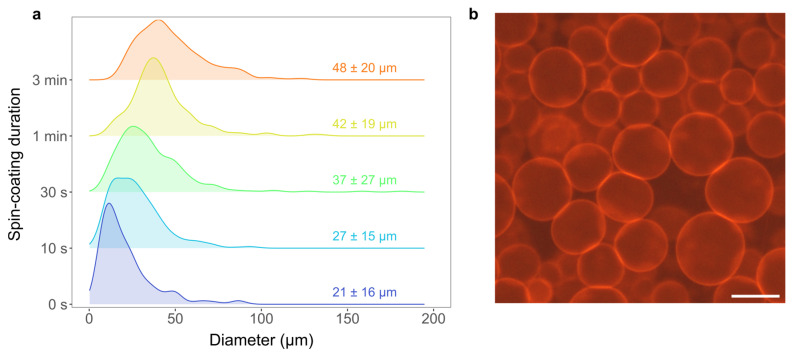
Size distribution densities of GUVs produced using the new protocol. (**a**) Comparison of size distributions for five spin-coating durations ranging from 0 s to 3 min. Each distribution represents 300 randomly selected vesicles from three independent samples (100 vesicles per sample). If the sample did not contain 100 vesicles, all vesicles from that sample were taken into account. (**b**) Fluorescence microscopy image of GUVs produced using the new protocol with 3 min of spin coating. The scale bar represents 50 m.

Using different durations of spin-coating, ranging from 0 s to 3 min, we tested the effect of spin-coating duration on the efficiency of GUV formation (Figure 5a). The 0 s case involves no spin-coating, but the LUV suspension is instead simply dropped onto the plasma-cleaned electrode and the excess liquid is shaken off after 10 s. We performed three experiments for every duration of spin-coating. The obtained mean size and standard deviation ranged from 21 ± 16 m to 48 ± 20 m for the 0 s and 3 min groups of samples, respectively (Figure 5a). The average size shows a clear dependence on spin-coating duration with longer drying being more favorable for production of GUV populations with larger size and yield. 

Compared to previous research from our group which dealt with optimization of GUV electroformation from a dry lipid film [21,22], the sizes and quality of vesicles determined using fluorescence microscopy images were similar or better for both durations of spin-coating, confirming the benefits of our protocol in that regard as well (Figure 5b).

### 3.3. GUVs Grown from Different Lipid Mixtures

**Figure 6:**
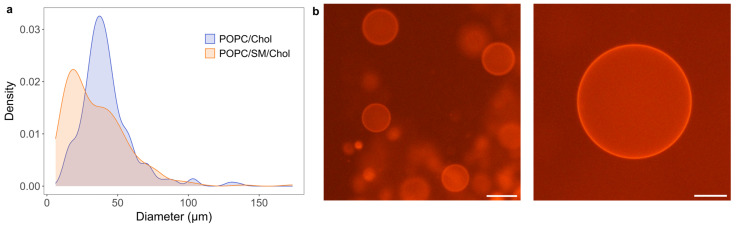
(**a**) Comparison of size distribution densities for GUVs produced from 1/1 POPC/Chol and 1/1/1 POPC/SM/Chol mixtures with a spin-coating duration of 1 min. (**b**) Fluorescence microscopy images of GUVs for the POPC/SM/Chol mixture. The scale bar represents 50 µm.

Aside from the binary 1/1 POPC/Chol mixture, we have also produced GUVs from a ternary 1/1/1 POPC/SM/Chol mixture which is important for researchers studying lipid rafts [40,41,42]. The hydrodynamic diameter of SM-containing LUVs was measured at 136 nm with a polydispersity index of 0.11. Using a spin-coating duration of 1 min, we have successfully produced GUVs from this mixture as well. We obtained an average diameter of 35 ± 21 . Comparing this to the corresponding result for the POPC/Chol mixture of 42 ± 19 m, we can see that the vesicles were smaller after inclusion of SM (*p* = 2) (Figure 6). This is consistent with our previous research, showing that inclusion of SM in the lipid mixture makes it harder to produce GUVs with large average size and yield [21,22]. The average size of the SM-containing GUV population is also on par with the best results obtained using the conventional lipid film deposition methods [21].

We observed no lateral phase separation in GUVs produced from the ternary POPC/SM/Chol mixture (Figure 6b). Depending on the membrane model type and method used, different phase diagrams have been reported for similar mixtures [41,42,43]. A study performed on GUVs produced from a 1/1/1 mixture of POPC/palmitoyl sphingomyelin/Chol showed that they should undergo phase separation at a transition temperature of approximately 20 °C [42]. Therefore, the temperature at which the GUV microscopy was performed might have been too high to observe the separation. Moreover, they used palmitoyl sphingomyelin and not egg sphingomyelin, so that could also affect the expected phase behavior. Additionally, all mentioned studies used a protocol which contained a dry film step [41,42,43], so the Chol content specified in the diagrams might have actually been lower due to demixing.

The proposed protocol should help reduce the Chol demixing artifact and improve the ability to produce high quality GUV populations under different conditions and from different lipid mixtures. However, a tradeoff seems to be involved. On one hand, in order to increase the certainty that Chol will not crystallize, the lipid film should remain as wet as possible. On the other hand, we have shown that shorter drying times result in lower yields and smaller average GUV diameters. If the lipid mixture contains no Chol, or small quantities of Chol, there is no reason not to dry the lipid film. However, if that is not the case, Chol demixing will certainly be an issue. The RSE technique is included to prevent Chol demixing during preparation of the MLV suspension, and keeping the lipid film damp should prevent Chol demixing during lipid film deposition. It should be noted that we have not quantified the exact Chol content in produced GUVs. However, to address this in the future, we plan to perform confocal fluorescence microscopy experiments using two fluorescent dyes—a phospholipid and a Chol analogue. Comparison of fluorescence intensity profiles between GUVs grown using the conventional and our newly developed protocol should reveal the level of demixing. Moreover, if our protocol reduced the demixing artifact, pure Chol bilayer domains should form between 50 and 66 mol% of Chol in the initial lipid mixture.

We have also successfully produced high quality POPC GUVs by eliminating the RSE step of the protocol and using the gentle hydration approach to produce MLVs instead (Supplementary Figure S1). Additionally, in the previous subsection, we have shown that spin-coating can be avoided as well by simply dropping the LUV suspension on the plasma-cleaned electrode and shaking off the excess liquid after 10 s. Alternatively, the LUV suspension could simply be directly deposited into a chamber with a plasma-cleaned electrode. However, this approach hampers subsequent microscopy due to a strong signal from LUVs that remain in the solution. Therefore, sample dilution or an additional purification step are required to adequately visualize the results. Both approaches reduce the yield and size of the obtained GUV population.

We believe that this new improved electroformation protocol will allow us to successfully study models of eye lens fiber cell membranes with their very high Chol content [32,33,44,45,46]. Furthermore, both using aqueous solutions and treating the electrodes with plasma have proven beneficial for electroformation efficiency, allowing for production of GUVs with charged lipids and solutions containing high ion concentrations [10,30]. Since it avoids organic solvents and lipid film drying, the protocol could also be adapted for protein reconstitution into GUVs.

## 4. Conclusions

We introduced a new improved electroformation protocol which bypasses the dry lipid film phase of the traditional approach by combining the plasma-cleaning, RSE, and spin-coating techniques. The protocol consists of 6 main steps:

1. The lipid solution is prepared from chloroform dissolved lipid stocks.
2. The obtained solution is mixed with deionized water and RSE is then used to obtain the MLV suspension.
3. MLVs are extruded by passing the solution through a polycarbonate filter in order to produce a homogeneous LUV solution.
4. The ITO electrodes which were stored in deionized water are cleaned by swabbing with ethanol moistened wipes and then plasma cleaned for additional cleaning and surface hydrophilization.
5. The LUV suspension is deposited onto the hydrophilic ITO electrode surface and spin-coated to produce a lipid film. The film is created due to vesicles rupturing in contact with the hydrophilic surface.
6. The coated electrode is used in construction of the electroformation chamber which is subsequently connected to an alternatincg current source in order to produce GUVs.

Previous studies have shown that electroformation from damp lipid films increases the compositional uniformity of the resulting GUV population and reduces the artifactual Chol demixing. Compared to the earlier damp lipid film protocol, our method significantly decreases the preparation time by eliminating the 24 h high-humidity drying phase and replacing it with a short duration of spin-coating. Furthermore, compared to the drop-deposition method, spin-coating can lead to higher experiment reproducibility. We believe that this new improved electroformation protocol will allow us to successfully study the physical properties, lateral organization and domain function of cell membranes with very high Chol such as the eye lens fiber cell membranes [32,33,44,45,46]. Additionally, both using aqueous solutions and treating the electrodes with plasma have proven beneficial for electroformation efficiency, allowing for production of GUVs with charged lipids and solutions containing high ion concentrations The protocol could also be adapted for protein insertion into GUVs with reduced protein denaturation due to the avoidance of organic solvents and lipid film drying.

## Data Availability

The data presented in this study are available upon reasonable request from the corresponding author.

## Supplementary Materials

The following supporting information can be downloaded at: https://www.mdpi.com/article/10.3390/membranes13030352/s1, Figure S1: Size distribution density for GUVs produced from POPC with a spin-coating duration of 2 min. The lipid concentration was 3 mg/mL.

## References

[1] Rideau E., Dimova R., Schwille P., Wurm F.R., Landfester K. (2018). Liposomes and Polymersomes: A Comparative Review towards Cell Mimicking. Chem. Soc. Rev..

[2] Reeves J.P., Dowben R.M. (1969). Formation and Properties of Thin-Walled Phospholipid Vesicles. J. Cell. Physiol..

[3] Rodriguez N., Pincet F., Cribier S. (2005). Giant Vesicles Formed by Gentle Hydration and Electroformation: A Comparison by Fluorescence Microscopy. Colloids Surf. B Biointerfaces.

[4] Angelova M.I., Dimitrov D.S. (1986). Liposome Electroformation. Faraday Discuss. Chem. Soc..

[5] Dimitrov D.S., Angelova M.I. (1988). Lipid Swelling and Liposome Formation Mediated by Electric Fields. J. Electroanal. Chem..

[6] Grusky D.S., Bhattacharya A., Boxer S.G. (2023). Examining Compositional Variability of Giant Unilamellar Vesicles via Secondary Ion Mass Spectrometry. Biophys. J..

[7] Boban Z., Mardešić I., Subczynski W.K., Raguz M. (2021). Giant Unilamellar Vesicle Electroformation: What to Use, What to Avoid, and How to Quantify the Results. Membranes.

[8] Veatch S.L. (2007). Electro-Formation and Fluorescence Microscopy of Giant Vesicles with Coexisting Liquid Phases. Lipid Rafts.

[9] Morales-Penningston N.F., Wu J., Farkas E.R., Goh S.L., Konyakhina T.M., Zheng J.Y., Webb W.W., Feigenson G.W. (2010). GUV Preparation and Imaging: Minimizing Artifacts. Biochim. Biophys. Acta-Biomembr..

[10] Pott T., Bouvrais H., Méléard P. (2008). Giant Unilamellar Vesicle Formation under Physiologically Relevant Conditions. Chem. Phys. Lipids.

[11] Baykal-Caglar E., Hassan-Zadeh E., Saremi B., Huang J. (2012). Preparation of Giant Unilamellar Vesicles from Damp Lipid Film for Better Lipid Compositional Uniformity. Biochim. Biophys. Acta-Biomembr..

[12] Méléard P., Bagatolli L.A., Pott T. (2009). Giant Unilamellar Vesicle Electroformation. From Lipid Mixtures to Native Membranes Under Physiological Conditions. Methods Enzymol..

[13] Witkowska A., Jablonski L., Jahn R. (2018). A Convenient Protocol for Generating Giant Unilamellar Vesicles Containing SNARE Proteins Using Electroformation. Sci. Rep..

[14] Collins M.D., Gordon S.E. (2013). Giant Liposome Preparation for Imaging and Patch-Clamp Electrophysiology. J. Vis. Exp..

[15] Bhatia T., Husen P., Brewer J., Bagatolli L.A., Hansen P.L., Ipsen J.H., Mouritsen O.G. (2015). Preparing Giant Unilamellar Vesicles (GUVs) of Complex Lipid Mixtures on Demand: Mixing Small Unilamellar Vesicles of Compositionally Heterogeneous Mixtures. Biochim. Biophys. Acta-Biomembr..

[16] Girard P., Pécréaux J., Lenoir G., Falson P., Rigaud J.L., Bassereau P. (2004). A New Method for the Reconstitution of Membrane Proteins into Giant Unilamellar Vesicles. Biophys. J..

[17] Estes D.J., Mayer M. (2005). Electroformation of Giant Liposomes from Spin-Coated Films of Lipids. Colloids Surf. B Biointerfaces.

[18] Oropeza-Guzman E., Riós-Ramírez M., Ruiz-Suárez J.C. (2019). Leveraging the Coffee Ring Effect for a Defect-Free Electroformation of Giant Unilamellar Vesicles. Langmuir.

[19] Berre L., Chen Y., Baigl D. (2009). From Convective Assembly to Landau-Levich Deposition of Multilayered Phospholipid Films of Controlled Thickness. Langmuir.

[20] Taylor P., Xu C., Fletcher P.D.I., Paunov V.N. (2003). A Novel Technique for Preparation of Monodisperse Giant Liposomes. Chem. Commun..

[21] Boban Z., Mardešić I., Subczynski W.K., Jozić D., Raguz M. (2022). Optimization of Giant Unilamellar Vesicle Electroformation for Phosphatidylcholine/Sphingomyelin/Cholesterol Ternary Mixtures. Membranes.

[22] Boban Z., Puljas A., Kovač D., Subczynski W.K., Raguz M. (2020). Effect of Electrical Parameters and Cholesterol Concentration on Giant Unilamellar Vesicles Electroformation. Cell Biochem. Biophys..

[23] Politano T.J., Froude V.E., Jing B., Zhu Y. (2010). AC-Electric Field Dependent Electroformation of Giant Lipid Vesicles. Colloids Surf. B Biointerfaces.

[24] Billerit C., Jeffries G.D.M., Orwar O., Jesorka A. (2012). Formation of Giant Unilamellar Vesicles from Spin-Coated Lipid Films by Localized IR Heating. Soft Matter.

[25] Huang J., Buboltz J.T., Feigenson G.W. (1999). Maximum Solubility of Cholesterol in Phosphatidylcholine and Phosphatidylethanolamine Bilayers. Biochim. Biophys. Acta-Biomembr..

[26] Raguz M., Kumar S.N., Zareba M., Ilic N., Mainali L., Subczynski W.K. (2019). Confocal Microscopy Confirmed That in Phosphatidylcholine Giant Unilamellar Vesicles with Very High Cholesterol Content Pure Cholesterol Bilayer Domains Form. Cell Biochem. Biophys..

[27] Buboltz J.T. (2009). A More Efficient Device for Preparing Model-Membrane Liposomes by the Rapid Solvent Exchange Method. Rev. Sci. Instrum..

[28] Mainali L., Raguz M., Subczynski W.K. (2013). Formation of Cholesterol Bilayer Domains Precedes Formation of Cholesterol Crystals in Cholesterol/Dimyristoylphosphatidylcholine Membranes: EPR and DSC Studies. J. Phys. Chem. B.

[29] Mainali L., Pasenkiewicz-Gierula M., Subczynski W.K. (2020). Formation of Cholesterol Bilayer Domains Precedes Formation of Cholesterol Crystals in Membranes Made of the Major Phospholipids of Human Eye Lens Fiber Cell Plasma Membranes. Curr. Eye Res..

[30] Li Q., Wang X., Ma S., Zhang Y., Han X. (2016). Electroformation of Giant Unilamellar Vesicles in Saline Solution. Colloids Surf. B Biointerfaces.

[31] Hardy G.J., Nayak R., Zauscher S. (2013). Model Cell Membranes: Techniques to Form Complex Biomimetic Supported Lipid Bilayers via Vesicle Fusion. Curr. Opin. Colloid Interface Sci..

[32] Mainali L., Raguz M., O’Brien W.J., Subczynski W.K. (2014). Properties of Membranes Derived from the Total Lipids Extracted from Clear and Cataractous Lenses of 61–70-Year-Old Human Donors. Eur. Biophys. J..

[33] Mainali L., Raguz M., O’Brien W.J., Subczynski W.K. (2013). Properties of Membranes Derived from the Total Lipids Extracted from the Human Lens Cortex and Nucleus. Biochim. Biophys. Acta-Biomembr..

[34] Mason R.P., Tulenko T.N., Jacob R.F. (2003). Direct Evidence for Cholesterol Crystalline Domains in Biological Membranes: Role in Human Pathobiology. Biochim. Biophys. Acta-Biomembr..

[35] Subczynski W.K., Pasenkiewicz-Gierula M. (2020). Hypothetical Pathway for Formation of Cholesterol Microcrystals Initiating the Atherosclerotic Process. Cell Biochem. Biophys..

[36] Herold C., Chwastek G., Schwille P., Petrov E.P. (2012). Efficient Electroformation of Supergiant Unilamellar Vesicles Containing Cationic Lipids on ITO-Coated Electrodes. Langmuir.

[37] Schindelin J., Arganda-Carreras I., Frise E., Kaynig V., Longair M., Pietzsch T., Preibisch S., Rueden C., Saalfeld S., Schmid B. (2012). Fiji: An Open-Source Platform for Biological-Image Analysis. Nat. Methods.

[38] R Development Core Team (2020). R: A Language and Environment for Statistical Computing.

[39] Ong S., Chitneni M., Lee K., Ming L., Yuen K. (2016). Evaluation of Extrusion Technique for Nanosizing Liposomes. Pharmaceutics.

[40] Kusumi A., Fujiwara T.K., Tsunoyama T.A., Kasai R.S., Liu A., Hirosawa K.M., Kinoshita M., Matsumori N., Komura N., Ando H. (2020). Defining Raft Domains in the Plasma Membrane. Traffic.

[41] de Almeida R.F.M., Fedorov A., Prieto M. (2003). Sphingomyelin/Phosphatidylcholine/Cholesterol Phase Diagram: Boundaries and Composition of Lipid Rafts. Biophys. J..

[42] Veatch S.L., Keller S.L. (2005). Miscibility Phase Diagrams of Giant Vesicles Containing Sphingomyelin. Phys. Rev. Lett..

[43] Ionova I.V., Livshits V.A., Marsh D. (2012). Phase Diagram of Ternary Cholesterol/Palmitoylsphingomyelin/Palmitoyloleoyl-Phosphatidylcholine Mixtures: Spin-Label EPR Study of Lipid-Raft Formation. Biophys. J..

[44] Li L.K., So L., Spector A. (1985). Membrane Cholesterol and Phospholipid in Consecutive Concentric Sections of Human Lenses. J. Lipid Res..

[45] Li L.-K., So L., Spector A. (1987). Age-Dependent Changes in the Distribution and Concentration of Human Lens Cholesterol and Phospholipids. Biochim. Biophys. Acta-Lipids Lipid Metab..

[46] Deeley J.M., Mitchell T.W., Wei X., Korth J., Nealon J.R., Blanksby S.J., Truscott R.J.W. (2008). Human Lens Lipids Differ Markedly from Those of Commonly Used Experimental Animals. Biochim. Biophys. Acta-Mol. Cell Biol. Lipids.

