# Supplementary material for: Electroformation of Giant Unilamellar Vesicles from Damp Lipid Films Formed by Vesicle Fusion
Source: Membranes (Basel). 2023 Mar 18;13(3):352. doi: 10.3390/membranes13030352 (PMC10059949; doi:10.3390/membranes13030352)
Supplement: Supplementary file 1 [file membranes-13-00352-s001.zip › membranes-2264151-supplementary.pdf]

Supplementary Material

Electroformation of Giant Unilamellar Vesicles from Damp Lipid Films Formed by Vesicle Fusion

Zvonimir Boban [1,2], Ivan Mardešić [1,2], Sanja Perinović Jozić [3], Josipa Šumanovac [4], Witold Karol Subczynski [5] and Marija Raguz [1,*]

[1] Department of Medical Physics and Biophysics, University of Split School of Medicine, 21000 Split, Croatia; zvonimir.boban@mefst.hr (Z.B.); imardesi@mefst.hr (I.M.); marija.raguz@mefst.hr (M.R.)
[2] Faculty of Science, University of Split, Doctoral Study of Biophysics, 21000 Split, Croatia
[3] Department of Organic Technology, Faculty of Chemistry and Technology, University of Split, 21000 Split, Croatia
[4] Faculty of Science, University of Split, Department of Physics, 21000 Split, Croatia
[5] Department of Biophysics, Medical College of Wisconsin, Milwaukee, WI 53226, USA; subczyn@mcw.edu
* Correspondence: marija.raguz@mefst.hr; Tel.: +385-9876-8819

Supplementary Figure S1 shows the size density distribution of GUVs produced from POPC. Instead of RSE, the gentle hydration method was used to produce MLVs. All other steps were the same as in other experiments. The average GUV diameter (mean ± sd) was 55 ± 31 μm, with many GUVs larger than 100 μm and even as large as 300 μm being produced. There is no need to use the RSE method if no Chol, or small amount of Chol is present in the mixture. However, if that is not the case, since the gentle hydration protocol also involves a lipid film drying step, the Chol demixing will certainly be an issue. The RSE technique is included to assure that the Chol concentration in initial MLVs is the same as the concentration of Chol in the initial mixture of lipids dissolved in an organic solvent.

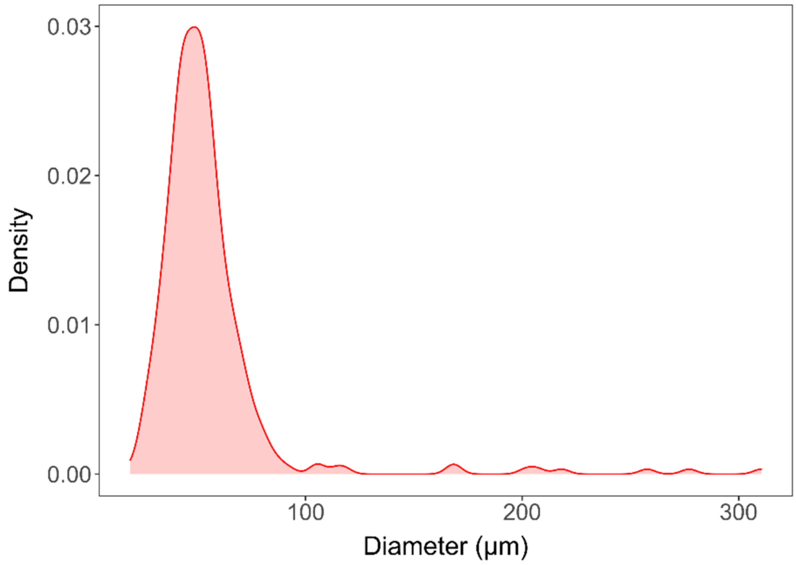

Figure S1. Size distribution density for GUVs produced from POPC with a spin-coating duration of 2 min. The lipid concentration was 3 mg/mL.
